# The Role of CYP450 Drug Metabolism in Precision Cardio-Oncology

**DOI:** 10.3390/ijms21020604

**Published:** 2020-01-17

**Authors:** Olubadewa A. Fatunde, Sherry-Ann Brown

**Affiliations:** 1 Department of Medicine, University of Texas Health Science Center at Tyler–CHRISTUS Good Shepherd Medical Center, Longview, TX 75601, USA; 2 Department of Cardiovascular Diseases, Mayo Clinic, Rochester, MN 55905, USA

**Keywords:** CYP450, drug metabolism, precision Cardio-Oncology, precision medicine, systems medicine

## Abstract

As many novel cancer therapies continue to emerge, the field of Cardio-Oncology (or onco-cardiology) has become crucial to prevent, monitor and treat cancer therapy-related cardiovascular toxicity. Furthermore, given the narrow therapeutic window of most cancer therapies, drug-drug interactions are prevalent in the cancer population. Consequently, there is an increased risk of affecting drug efficacy or predisposing individual patients to adverse side effects. Here we review the role of cytochrome P450 (CYP450) enzymes in the field of Cardio-Oncology. We highlight the importance of cardiac medications in preventive Cardio-Oncology for high-risk patients or in the management of cardiotoxicities during or following cancer treatment. Common interactions between Oncology and Cardiology drugs are catalogued, emphasizing the impact of differential metabolism of each substrate drug on unpredictable drug bioavailability and consequent inter-individual variability in treatment response or development of cardiovascular toxicity. This inter-individual variability in bioavailability and subsequent response can be further enhanced by genomic variants in CYP450, or by modifications of CYP450 gene, RNA or protein expression or function in various ‘omics’ related to precision medicine. Thus, we advocate for an individualized approach to each patient by a multidisciplinary team with clinical pharmacists evaluating a treatment plan tailored to a practice of precision Cardio-Oncology. This review may increase awareness of these key concepts in the rapidly evolving field of Cardio-Oncology.

## 1. Introduction

**Figure 1:**
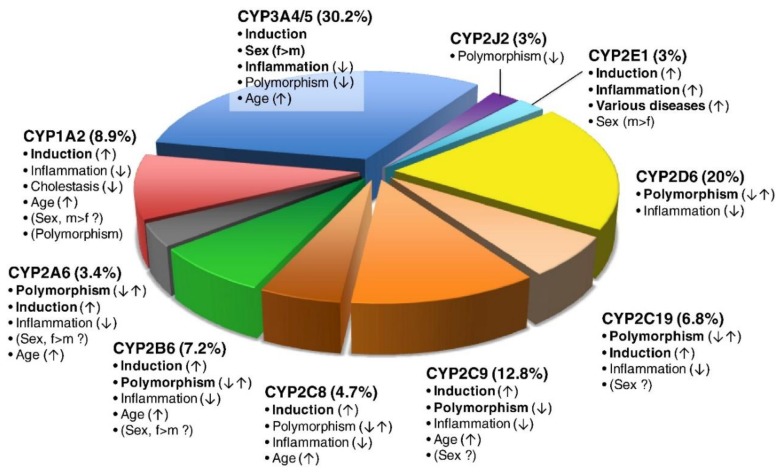
The pie chart depicts the various P450 isoforms, the percentage of clinically used drugs metabolized by the isoform and factors inducing or inhibiting the respective P450 enzyme, thereby influencing variability. The most important factors influencing variability are in bold, with a vertical arrow indicating increased activity (↑), decreased activity (↓) or both (↑↓). Biologic sex (female (f) or male (m)) and rarely polymorphism (CYP1A2) can be of controversial significance. In total, 248 CYP-related drug metabolism pathways were analyzed (excluding chemicals and endogenous substrates). Used with permission [1].

Cardio-Oncology is an emerging field that sits at the interface of Cardiology and Oncology and has close relationships with primary care specialties. A variety of oncology drugs can injure the cardiovascular system, causing various forms of cardiovascular toxicities. Further, cardiology drugs are widely used by the general population and by individuals with cancer. Many of these drugs are also frequently used for preventive cardioprotection or for the management of cardiotoxicity that has already occurred. In this review, we highlight several cytochrome P450 (CYP450) enzymes relevant to Cardio-Oncology (Figure 1). We classify drugs as CYP450 substrates, inducers or inhibitors, with an explanation of the three types of drug-enzyme interaction. Drug-drug interactions between Oncology and Cardiology drugs mediated by CYP450 enzymes are also surveyed. In addition, we discuss the fact that differential metabolism of each substrate drug in each specific individual can determine bioavailability. Examples from precision Cardio-Oncology are integrated to illustrate that inter-individual bioavailability can be further enhanced by genomic variation in CYP450 enzymes. Some variants enhance enzyme activity, while others do just the opposite. This helps to determine the level of drug available in the body. Not only genomic variants but also other modifications of the enzyme gene, RNA or protein, including those due to gene-environment interactions, can alter drug levels and individual response in precision medicine. We hope that this review can assist multidisciplinary teams in Cardio-Oncology with difficult drug-related decisions relevant to metabolism and bioavailability.

## 2. CYP450 Class of Enzymes

The CYP450 monooxygenase system consists of a family of enzymes that metabolize a variety of medications relevant to Cardiology and Oncology. The CYP450 enzymes are primarily located in the liver but can also be found in the small intestines, lungs, kidneys and even the heart [1,2,3,4]. These enzymes are responsible for the first pass metabolism and largely explain the higher pharmacokinetic variability of oral drugs compared to intravenous medications [5,6]. Their etymology derives from their intracellular, membrane-bound localization (i.e., cyto-), with a heme pigment forming part of the protein (i.e., chrome). The heme portion of the enzymes absorbs light at a maximum wavelength of 450 nm when complexed with carbon monoxide in the reduced state. In humans, more than 100 collective genes and pseudogenes encode over 50 CYP450 enzymes. CYP1A2, CYP2C9, CYP2C19, CYP2D6 and CYP3A4/5 metabolize over 90% of the substrate drugs and are the most extensively studied CYP450 enzymes [1,2,3,7] (Table 1). 

Drug metabolism in the liver occurs in three major steps: hepatic (transporter-mediated) uptake, phase I reactions and phase II reactions. Hepatic uptake is responsible for a trivial amount of pharmacokinetic variability. In phase I reactions, the CYP450 enzymes oxidize, reduce or hydrolyze their substrates, resulting in loss of pharmacological activity or activation of prodrugs [3]. In phase II reactions, non-CYP450 enzymes conjugate phase I products by adding glucuronide, acetyl, methyl or sulphate groups to form usually inactive polar derivatives for renal or biliary elimination [3,8].

### 2.1. Phase I Enzymes

The most predominant CYP450 enzyme class involved in phase I reactions is the CYP3A family. CYP3A drugs metabolize 45–60% of all drugs currently in use [9,10], with CYP3A4 representing the most common allele. CYP3A4 is predominantly found in the liver [11,12] and intestines [13,14] and can also be found in the stomach, brain, breast and prostate [15]. A second phase I enzyme CYP3A5 is present in the liver and small intestine of 25–30% of individuals [1,3]. A third enzyme CYP3A7 is present predominantly in fetuses (50% of total expression), with expression typically shifting to CYP3A4 in adulthood [1]. CYP1A2 is constitutively expressed primarily in the liver, in significant quantities, measuring up to 16% of the total hepatic P450 pool in some individuals [1]. CYP2C9 is the second most common CYP450 enzyme found in the liver and extrahepatic tissues such as the intestines and endothelial cells; many CYP2C9 substrates (e.g., warfarin) have narrow therapeutic indices, requiring careful monitoring in patients taking these drugs. CYP2C19 is expressed in the liver and kidneys and is responsible for the metabolism clopidogrel, a drug commonly used in cardiology and with relevance for Cardio-Oncology; genetic variation is often associated with adverse drug effects. The CYP2D6 enzyme is primarily expressed outside of the liver and metabolizes approximately 15–25% of drugs from all therapeutic areas, including Cardiology (e.g., beta-blockers, antiarrhythmics) and Oncology (e.g., tamoxifen). There is substantial inter-individual variability with hepatic CYP450 enzyme activity, ranging from 30- to 40-fold variation for collective CYP3A enzymes [16,17,18,19] to 100-fold variation for CYP2D6 [16,19].

### 2.2. Phase II Enzymes

Phase II enzymes are non-CYP450 proteins that can indirectly exert influence on CYP450 enzyme activity. The most commonly occurring phase II reactions are glucoronidation and sulfonation (or sulfurylation), which are catalyzed by the enzymes uridine diphosphate glucoronysltransferase (UGTs) and sulfotransferases (SULTs), respectively. These enzymes are located throughout the gastrointestinal and genitourinary tracts. 

The UGTs catalyze transfer of glucuronic acid to onto oxygen, nitrogen or sulfur on substrate drugs. Substrates range from endogenous substances (e.g., bilirubin, estradiol, serotonin) to exogenous substances (e.g., propofol, morphine). Interindividual variability in levels of UGT, stemming from a patient’s age, sex, presence of enzyme inhibitors/inducers can contribute to drug-induced toxicity (slow metabolism) or ineffectual drug levels (rapid metabolism) [20]. 

Toxic drug metabolites of UGTs levels can lead to additive toxicity with P450 (phase I) drug-drug interactions. For example, the glucoronated products of gemfibrozil can inactivate CYP2C8, causing toxic levels of statins and significant rhabdomyolysis [21]. Likewise, clopidogrel can also inactivate CYP2C8, resulting in toxic levels of gemfibrozil [22]. Finally, UGT1 levels have been shown to inversely associate with development of a number of cancers (i.e., colon cancer, breast, bladder and biliary) in conditional UGT1 knockout mice [23]. 

Sulfonation reactions result in increased hydrophilicity and (usually) decreased pharmacological activity or inactivation of certain endogenous substances, such as thyroid hormones, steroids and monoamine transmitters. Inhibition of sulfonation by some compounds or metabolites can increase the toxicity of these substances [24,25]. Conversely, sulfonation can also bioactivate some substrates. This can result in a benign, more metabolically active form (e.g., minoxidil, morphine) [25] or can produce certain toxic metabolites, thereby increasing drug toxicity (e.g., tamoxifen) [24,26]. There are three main SULTs supergene families in humans—SULT1, SULT2, SULT4 [24,25,26]. SULT1A is most concentrated in the liver and has also been found in the kidney, lung, brain and gastrointestinal and genitourinary systems. The extensive expression of SULT1A1 and SULT1A3 in the intestines and lungs suggest they may play a role in extrahepatic drug detoxification and metabolism. SULT1B functions in thyroid hormone metabolism. SULT2A and SULT2B family are active in the metabolism of steroids and bile acids and are present throughout the body. Notably, products of sulfonation reactions catalyzed by various SULT enzymes (e.g., 1A1, 1A2, 1A3, 1C2, 1C4 and 2A1) can result in chemically reactive intermediate compounds that bind DNA, eliciting mutagenicity and carcinogenicity [25]. Interindividual variation in human sulfotransferase activity varies from 5- to 36-fold, largely explained by single nucleotide polymorphisms (SNPs) in the coding regions of SULT genes. This variation can play a complementary role to phase I reactions (largely catalyzed by P450 enzymes) in determining an individual’s response to therapeutics. 

### 2.3. Substrates, Inducers and Inhibitors

Drugs that interact with the CYP450 enzymes can be divided into three categories: substrates, inhibitors and inducers. Substrates are drugs upon which specific CYP450 enzyme acts. Inducers are drugs that increase enzyme activity. Inhibitors are drugs that decrease enzyme activity. Inhibitors compete with other drugs (typically substrates) for enzyme active sites, therefore altering the optimal level of a given substrate drug in the plasma. This alters the intended drug pharmacokinetics, rendering many prodrugs ineffective or conversely, potentially raising other drugs’ plasma concentrations to toxic levels. A strong inhibitor is defined as one that increases plasma AUC substrate values greater than 5-fold or decreases substrate clearance to more than 80% of normal levels. A moderate inhibitor causes a greater than 2-fold increase in the plasma AUC values or a 50–80% decrease in drug clearance. A weak inhibitor causes a greater than 1.25-fold increase in plasma AUC values or a 20–50% decrease in drug clearance. Drugs commonly used in cardiology fall into all three categories (substrates, inducers and inhibitors) (Table 2) and can potentially interact with oncology drug substrates; the converse is also true. Being mindful of drug-drug interactions due to CYP450 activity related to substrates, inducers and inhibitors may help protect the hearts of patients undergoing cancer therapies. 

## 3. Drug-Drug Interactions 

Drug-drug interactions are fairly common in the oncologic patient with cardiac disease (Table 2 and Table 3). In one study [42], 16% of patients receiving oral antineoplastic agents developed at least one major drug-drug interaction. This is of particular concern, given the narrow therapeutic window of many antineoplastic agents and some cardiology medications. In another study, a range of drug-drug interactions involving chemotherapeutic and common cardiac medications resulting from either pharmacokinetic (PK) interactions, pharmacodynamic (PD) interactions or a combination of the two was described [3,43]. The most common PK interactions in oncology involve the CYP450 enzymes and the efflux pump P-glycoprotein located in the intestine [43,44]. In essence, PK interactions describe the body’s effect on a drug or substance, especially its absorption, distribution, metabolism or elimination [44,45]. On the other hand, PD interactions describe a drug’s effect on the body. Drug-drug interactions in this arena are due to often unintentional additive effects of two agents with similar molecular targets, resulting in toxicity. 

An example of a PD interaction in Cardio-Oncology occurs with concurrent use of beta-blockers (in Cardiology) and ceritinib/crizotinib (in Oncology); the latter is a combination chemotherapeutic drug used to treat metastatic (ALK-/ROS1-positive) non-small cell lung cancer. Co-administration of these medications can lead to symptomatic bradycardia, which can potentially be life-threatening [43]. Additionally, ceritinib/crizotinib can prolong the QT interval. Therefore, administration with other QT-prolonging medications that are often administered with chemotherapy, such as antiemetics, antibiotics and antidepressants, can potentially lead to malignant arrhythmias, including polymorphic ventricular tachycardia or ‘torsades de pointes.’ [43,46]. Consequently, beta-blockers or QT-prolonging medications should be used judiciously with ceritinib/crizotinib if co-administered with any drugs that inhibit CYP3A, as both ceritinib and crizotinib are extensively metabolized by CYP3A in the liver [47,48]. 

An example of a PK interaction in Cardio-Oncology involves the moderate inhibition of CYP3A4 by diltiazem/verapamil. When co-administered with chemotherapeutic agents metabolized by the same pathway, such as doxorubicin, imatinib or ibrutinib, this could lead to increased chemotherapy drug concentration. This can be accompanied by several adverse effects, including QT prolongation, gastrointestinal symptoms, shortness of breath, edema, chest pain, hepatotoxicity or bone marrow suppression [43]. This can be managed by using alternative medications for chronotropy or blood pressure control during the expected course of chemotherapy or appropriately decreasing the dose of administered chemotherapy if absolutely necessary [43,46]. 

Antiplatelet agents are a mainstay of atherosclerotic cardiovascular disease treatment and account for 40.4% of drug sales in cardiovascular disease [49]. PK interactions between antiplatelet agents and chemotherapeutics can alter the level of functioning of one or both drugs. For example, the chemotherapeutic combination agent enzalutamide/dasatinib can decrease the level of antiplatelet medication in the blood, causing disastrous/catastrophic consequences following cardiac catheterization [43]. When doxorubicin is administered with ticagrelor, CYP3A4 inhibition by ticagrelor can lead to an increase in doxorubicin exposure, placing patients at increased risk for known toxicities of the drug [43]. Based on the indicated chemotherapy regimen, the antiplatelet agent can usually be adjusted, taking into account patient characteristics. 

Anticoagulants are indicated for patients with malignancies who develop deep venous thromboses (DVT) or pulmonary emboli (PE), which complicate the clinical course of approximately 5–10% of all cancer patients [50]. In patients with malignancies who also have cardiovascular disease requiring anticoagulation (e.g., atrial fibrillation, mechanical valves, mechanical support devices), the number of concurrent medications can increase the risk of drug-drug interaction. Historically, warfarin has been the most commonly used anticoagulant and remains in frequent use due to familiarity, cost and patient preference. Most drug-drug interactions involving chemotherapeutic agents and warfarin are due to a reduction in warfarin metabolism, often from CYP450 inhibition, leading to increased risk of bleeding (Table 2) [43]. As warfarin operates via vitamin K inhibition, oncologic patients with numerous reasons for vitamin K deficiency, such as diarrhea from chemotherapy or radiation or antibiotics for infections due to immunosuppression, face an additionally increased risk of bleeding [43]. The current standard of care for management of cancer-associated venous thromboembolism (VTE) is low molecular weight heparin [51,52,53,54,55]. Direct oral anticoagulants (DOACs) are emerging as potentially equally efficacious alternatives to low molecular weight heparin, with ideal bioavailability and mode of administration (orally) [29]. However, DOACs should be used cautiously in certain populations at increased risk for bleeding complications (e.g., gastrointestinal malignancies, advanced age and frailty) [43,50]. It is of note that DOACs are variably metabolized by CYP450 enzymes (dabigatran 0%, edoxaban < 4%, apixaban 15%, rivaroxaban 66%) [29]. Therefore, in cancer patients concurrently treated with strong CYP3A4 inducers or inhibitors (Table 2), dabigatran (or possibly edoxaban) may become the DOAC of choice; such decision-making may benefit from close collaboration with a clinical pharmacist [29,56]. Nevertheless, both LMWH and DOACs have fewer drug-drug interactions than warfarin [43,56,57,58].

## 4. Precision Cardio-Oncology

### 4.1. Variability in Concentration and Activity

There can be wide variation in the concentration levels and activity of CYP450 between and within populations, as illustrated in the following examples. In the general population, there is up to 50-fold variation in the levels of CYP3A4 among individuals [15,59]. In a small study investigating the metabolism of cyclophosphamide in patients with lung, breast and gastrointestinal malignancies, the level of CYP2C19 was lower in patients with cancer compared to the general population [60]. Conversely, in a study investigating the role of miRNA in the regulation of CYP1B1, higher levels of CYP1B1 were noted particularly among individuals with estrogen-sensitive cancers [60,61]. Indeed, some CYP450 enzymes are preferentially upregulated by cancerous cells. For example, CYP1B1, mainly expressed in the ovary, uterus and breast tissue [61,62], is upregulated in malignant cells [63] to catalyze the metabolic activation of pro-carcinogens such as polycyclic aromatic hydrocarbons, aryl nitrate and 4-hydroxyestradiol [61]; 4-hydroxyestradiol is a catechol metabolite of 17β-estradiol, which generates free radicals, resulting in DNA damage [64,65]. Additionally, among patients with cancer, there can be wide variation in CYP450 enzyme activity.

### 4.2. Interindividual and Genetic Variability

Genetic variability, for example, of promoter or coding regions, may in part explain why different individuals have varying responses to the same drugs. Single nucleotide polymorphisms among CYP450 enzymes affect metabolism and therefore bioavailability of substrate drugs. Polymorphisms in CYP1A2 and CYP2B6 can result in decreased nicotine metabolism in smokers and have been associated with increased susceptibility to cancers and possibly atherosclerotic cardiovascular disease [1]. In particular, different combinations of alleles of CYP450 enzymes resulting in absent, low or high levels of enzymatic activity can lead to differential responses (e.g., toxicity, underdosing) to drug regimens, due to variable drug clearance [1,42,43].

### 4.3. Genomic Profiling

Genomic profiling in general characterizes an individual’s complement of genes [66]. Next generation sequencing and genome-wide association studies (GWAS), which correlate SNPs with disease phenotypes, have revolutionized the speed at which such information can be analyzed, investigated and translated into a component of clinical care [67]. Genomic profiling is most commonly utilized in oncology—particularly for breast, ovarian, colon and lung cancers [67]. The applicability in cardiology is increasing, from medication metabolism to treatment of cardiomyopathy and inherited arrhythmias [67]. Genomic profiling has a number of potential applications, including the study of genetic variations that influence individual response to drugs (i.e., pharmacogenomics), precision medicine and new modalities to diagnose and treat disease.

#### 4.3.1. Genomic Variation in CYP450

CYP450 genomics examine how modifications in the genes encoding the CYP450 complex affect enzyme function in metabolism and downstream drug therapeutic response. This has direct clinical applicability. This can be illustrated by examining two CYP450 enzymes relevant to Cardio-Oncology—CYP2C19 and CYP2D6 [7,16,68].

#### 4.3.2. Genomic Variation in CYP2C19

Over 50 CYP2C19 genomic variants have been identified [7]. The CYP2C19 gene is located on chromosome 10q24.1q24.3, is composed of nine exons and produces a medium-sized protein (55.93 kDa) from 490 amino acids [7]. Homozygosity for loss-of-function alleles confers poor metabolism, heterozygosity for loss-of-function (LOF) alleles confers intermediate metabolism, wild type alleles (*1/*1) confer what may be considered ‘normal’ metabolism and homozygosity for a gain-of-function (GOF) allele confers ultra-rapid metabolism [7]. The four major phenotypes listed above correspond to selected permutations of the most common CYP2C19 genetic variants (*1, *2, *3, *17) in the dose-response relationship (Table 3). The frequency of these phenotypes seems to differ with ethnicity. Approximately 2% of Europeans (the most widely studied population) are poor CYP2C19 metabolizers, while up to 20% of Asians are poor metabolizers [1,2,69], underscoring the need for further study in all populations. Poor or intermediate metabolism of clopidogrel may lead to persistently elevated platelet function in spite of treatment (i.e., high on-treatment platelet reactivity or HTPR) in individuals treated for acute coronary syndromes (ACS) [7]. These individuals remain at high-risk for ischemia, limited post-PCI myocardial flow and adverse cardiovascular outcomes (e.g., stent thrombosis, myocardial infarction, stroke and death) [7]. The most common SNPs responsible for the poor metabolizer phenotype result from premature stop codons due to the presence of Adenine in lieu of Guanine on nucleotide 681 of exon 5 (CYP2C19*2) and on nucleotide 636 of exon 3 (CYP2C19*2). While there are other alleles associated with CYP2C19 LOF (CYP2C19*4, *5, *6, *7, *8), they comprise less than one percent of the known CYP2C19 alleles [7]. Only one GOF variant (CYP2C19*17) has been identified.

**Table 3:** Anticipated CYP2C19 phenotypes corresponding to genotypes. Used with permission of Creative Commons [7], copyright 2019; and adapted from Reference [70], used with permission of John Wiley and Sons; copyright 2013.

Phenotype	Example Genotypes	Enzyme Activity
Ultra-rapid metabolizer (UM)	*1/*17 *17/*17	Normal or increased
Extensive metabolizer (EM)	*1/*1 (wild type)	Normal
Intermediate metabolizer (IM)	*1/*2 *1/*3 *2/*17 *3/17	Intermediate Likely intermediate Likely intermediate
Poor metabolizer (PM)	*2/*2 *3/*3 *2/*3	Low or absent

#### 4.3.3. Genomic Variation in CYP2D6

CYP2D6 phenotypes can also be categorized into 4 different groups based on enzyme activity: poor metabolizer, intermediate metabolizer, extensive metabolizer and ultra-rapid metabolizer [16,71,72,73,74,75]. The poor metabolizer phenotype is in part explained by genetic alterations, namely frame-shift mutations or splicing defects, yielding minimal expression of the CYP2D6 protein or a nonfunctional CYP2D6 protein [16]. There are over 100 CYP2D6 genomic variants, of which nearly half—through either decreased (CYP2D6*9, *17, *41, etc.) or no functional enzyme (CYP2D6 *4, *6, *15, etc.)—are phenotypically poor metabolizers [68]. The remaining known variants are phenotypically normal or as of yet undetermined. Less than 10% of the population possesses the 2D6 poor metabolizer phenotype and CYP2D6 metabolizes up to 20% of medications currently in use in the general population [16]. More research is needed to similarly characterize mechanisms behind the extensive and ultra-rapid CYP2D6 metabolizer phenotypes [16]. Further, translational and post-translational modifications may complement the contribution of genomics to interindividual variability. Among individuals with confirmed wild type (‘normal’) gene copies of CYP2D6, a remarkable extent of interindividual variability of phenotypic CYP2D6 activity was noted [16,76].

### 4.4. Systems Approach

**Figure 2:**
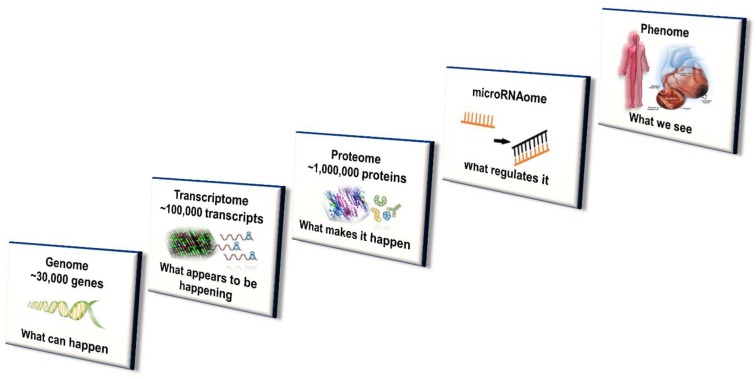
Multi-level systems-based approach to CYP450 expression, activity and regulation in Precision Cardio-Oncology. These multi-omics and additionally epigenomics, metabolomics, microbiomics and other personalization tools will likely be integrated in the future with mobile health, informatics and other emerging technologies for precision patient care relevant to Cardiology, Oncology and Cardio-Oncology.

**Figure 3:**
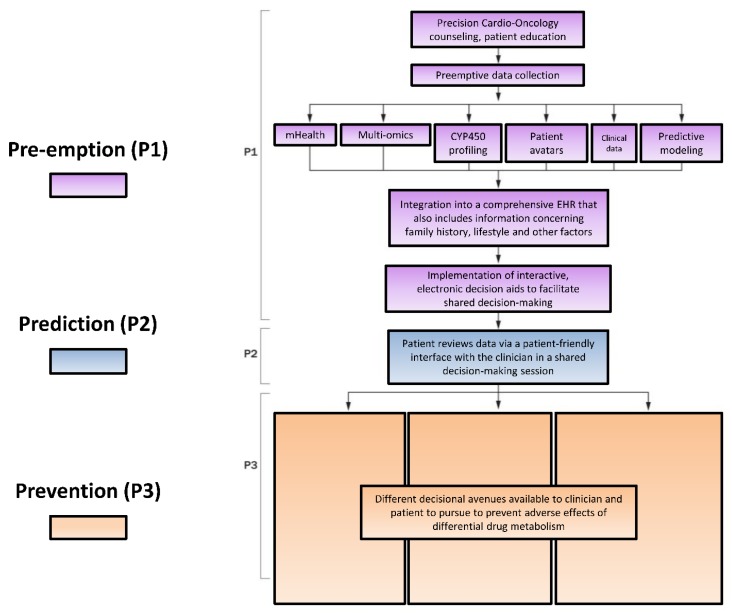
The P*3 Precision Medicine approach to individualizing therapy involving P450 enzymes. P1, Pre-empt: Pre-emption encompasses incorporation of elements of systems-based medicine, such as patients’ at-risk genetic polymorphisms, into the EHR seamlessly alongside clinical data. P2, Predict: Prediction describes the process of utilizing systems medicine data to estimate patients’ risk of developing cardiotoxicity. P3, Prevent: Prevention is proactively adjusting patients’ treatment plans based on their cardiovascular toxicity risk to prevent de novo damage or mitigate further damage from antineoplastic therapy. Adapted with permission [78]. CYP450 = cytochrome P450; EHR = electronic health record.

While genetic variation plays a distinct role, this does not completely account for interindividual differences in CYP450 enzyme concentration level and activity [7,16]. The contribution of variation in transcriptional regulators and (more recently) posttranscriptional protein modifications (especially alterations in microRNA) affecting CYP450 expression are gaining increasing recognition [15]. Further, ontogenic changes to CYP450 activity, due to xenobiotic exposure (e.g., phenobarbital) during early development and the early postnatal period, have been shown to have long-lasting effects in mouse models. Importantly, efforts to achieve both early diagnosis and optimal treatment of disease as well as prevent and mitigate cardiac adverse effects in oncology patients has led to study and potential application of multi-omic disciplines to the field of Cardio-Oncology [7,77]. Genomics, epigenomics, transcriptomics, proteomics, miRNAomics, metabolomics and microbiomics have the potential to more precisely guide the clinical management of these patients [7,78]. The integration of multi-omics with systems biology and incorporating disciplines such as mobile health (mHealth), pharmacogenomics, mathematical and computational modeling is depicted in Figure 2 and Figure 3 and has been well described [7,77,78].

#### 4.4.1. Transcriptomics

Transcriptomics or gene expression profiling, is the study of the complement of genes expressed, that is, mRNA [79]. Transcribed gene expression profiles can exhibit wide variability, depending on the physiologic stimuli. Genetic variation in promotor regions and transcriptional regulators of P450 enzymes can result in altered rates of gene expression and in turn, drug metabolism [16,80]. Approximately 10% of variation in target P450 enzyme expression is explained by shared hepatic P450s transcriptional regulators (e.g., hepatocyte nuclear factor (HNF) 4β, forkhead box proteins (FOX) A2 and A3 and pregnane X receptor (PXR) polymorphisms) [16,80].

#### 4.4.2. Epigenomics

Epigenomics is the study of physical modifications made to DNA molecules without alterations in DNA sequence. These modifications are most commonly but not limited to, acetylation and histone modification, methylation and transcription factor binding of DNA [7]. Both intrinsic and extrinsic factors can lead to epigenomic modifications of genes encoding CYP450 enzymes, thereby contributing to interindividual variability of therapeutic response [16,81]. Intrinsic factors include old age, congestive heart failure, cancer or pregnancy [7,82,83,84], while extrinsic factors include xenobiotics, tobacco smoke and air pollution [79]. Drugs can act as ligands to activate transcription factors, consequently inducing the expression of P450 genes. Phenobarbital and rifampin, for example, induce CYP2B6, CYP2C9 or CYP3A4 via activation of the nuclear receptors PXR or constitutive androstane receptor (CAR) [16]. It is hypothesized that epigenetic modifications in the promotor or coding region, in response to certain physiologic or pathophysiologic states, can either activate or silence certain CYP450 enzymes. For example, methylation of CpG motifs (CpG11, CpG12, CpG13) in the promoter region for CYP2C19 was associated with HTPR and increased risk of ischemic events on clopidogrel [7,85,86]. If an allele is preferentially silenced via epigenetic modifications in the coding region, the complementary allele will be predominantly expressed. Notably, DNA methylation and histone acetylation modification patterns have been found to be heritable in certain cases [79].

#### 4.4.3. Proteomics

Proteomics is the study of the complement of proteins and can also sometimes refer to chemical modifications (e.g., phosphorylation, acetylation, nitrosylation, etc.) undergone by proteins during and after translation, due to specific (patho)physiological states and their effects on protein structure and function [7,79]. These changes can occur hours following an exposure (e.g., drug administration). In one study, a decrease in peroxiredoxin-4 (a molecule associated with HTPR) was observed just 24 h following the administration of a loading dose of clopidogrel [7,87].

#### 4.4.4. Metabolomics

Proteins with enzymatic activity produce metabolites. Metabolomics is the study of all measurable metabolites resulting from biotransformation of compounds, often by CYP450 enzymes, corresponding to specific physiologic or pathophysiologic states [7,88]. Homeostasis of an individual’s metabolome is impacted by both intrinsic (e.g., resting metabolic rate, age, genotype, etc.) and extrinsic (e.g., xenobiotics, microbiome, surrounding environment, etc.) factors [7,84,87,88]. In cardioncology patients, metabolic analysis can identify a specific metabolic pattern—a metabotype—corresponding to specific (patho)physiologic states (e.g., heart failure, myocardial infarction, myocarditis, etc.) and may serve as a ‘yellow-brick road,’ of sorts, offering insights into the present state and potentially, the future arc of disease [7,77]. Indeed, changes in a patient’s metabolic profile over time may help identify predictive biomarkers associated with different clinical trajectories, while helping to identify an individual’s response to therapy (or lack thereof), preceding phenotypic changes, helping to guide therapy [7,77].

#### 4.4.5. Microbiomics

Microbiomics studies the impact of the human microbiome on disease states. As 70% of the millions of bacteria inhabiting the human body reside in the gut, the metabolic products of these bacteria play a role in modulating the body’s inflammatory response and impacting chronic disease [7]. For example, trimethylamine N-oxide (TMAO) is a product of dietary phosphatidylcholine and gut microbe metabolism of carnitine and betaine [7,88]. High levels of TMAO are independently associated with platelet hyperactivity and thrombosis of atherosclerotic plaques [7,89,90,91], increasing risk of cardiovascular events [7,89] and predicting all-cause mortality [7,91]. Additionally, aspirin has been observed to alter the composition of the gut microbiota. This likely accounts for its ability to abate the effect of TMAO on platelets [7,91,92].

#### 4.4.6. MicroRNAomics

The field of miRNAomics is quickly emerging and encompasses a diverse array of applications ranging from regulation of translation by noncoding miRNAs to small RNA therapies for challenging drug targets. Micro-RNAs (miRNAs) are a class of noncoding RNAs that play an important role in selective gene silencing, through which miRNAs regulate the diverse functions of target genes ranging from proliferation and apoptosis to differentiation. Specifically, mature miRNAs function by binding to complementary regions of transcripts (via the RNA induced silencing complex or RISC) to either (mechanically) prevent translation by the ribosome or cut target mRNAs into fragments, thereby precipitating their degradation inside the cell [15,61]. The former regulatory method of translational prevention is the predominant one in mammals (i.e., humans), while the latter method resulting in mRNA degradation is used in plants [15]. Over 1900 miRNAs have been identified in humans [15,93]. As a class, miRNAs are predicted to regulate up to 60% of the human genome [15,94,95], with miRNAs acting on one or multiple genes and single genes often targeted by one or more miRNAs [15,96].

The role of miRNAs in regulating CYP450 enzymes was first demonstrated nearly 15 years ago [61]. Since that time, the number of known miRNAs has nearly doubled and innovative techniques such as computer modeling (i.e., *in silico*) studies are being used to discover more miRNAs and determine their associated function at an exponential rate [15,80]. Data from such computer modeling, integrated with in vitro and in vivo techniques, revealed that translation of CYP3A4 mRNA is repressed by multiple miRNAs, including hsa-miR-577, hsa-miR-1, hsa-miR-532-3p and hsa-miR-627 [15]. Notably, another study found that specific miRNA profiles affected response to chemotherapy, with hsa-miR-577 and hsa-miR-1 significantly improving chemosensitivity following administration of chemotherapy for gastric cancer [15,97]. More studies are needed to confirm whether this increase in chemosensitivity is related to an inhibitory effect of hsa-miR-577 and hsa-miR-1 on CYP3A4 transcripts.

Various miRNAs are generally downregulated in various cancers, often to limit translation and promulgate the action of CYP450 enzymes promoting metabolic production of pro-carcinogens, facilitating cancer growth [61]. For example, expression of CYP1B1 is post-transcriptionally regulated by miR-27b. Studies have found that the downregulation [61] or actual deletion [98] of the 9q22.1 gene locus coding for miR-27b allows for the high expression of CYP1B1, which can facilitate tumor growth. While this inverse relationship of miR-27b downregulation and CYP1B1 upregulation expression mainly occurs in estrogen-sensitive malignancies, it is also found in urothelial or bladder cancers [61,98,99].

In addition, miR-34a has an inverse relationship with expression of CYP3A4 and CYP2C19 proteins, as well as transcription factors and other proteins that indirectly affect CYP450 levels or activity (e.g., AKR1D1 and SLC10A1) and is generally found at higher levels in females than males [80]. Females are also known to have higher CYP3A4 levels than males, suggesting miR-34a as a possible etiology for intersex differences in CYP3A4 activity [80]. The miR-34a is upregulated in several cancers, including hepatocellular cancer [80,100]. Given the inhibition of CYP3A4 and CYP2C19 by miR-34a, patients with high levels of miR-34a are potentially at an increased risk of drug toxicity. Conversely, miR148a increases levels of CYP3A4 and CYP2C19 and is downregulated in a number of cancers, including most of the lower gastrointestinal (GI) tumors, along with head & neck cancers, breast cancer, lung cancer and melanoma [80].

A myriad of miRNAs are being considered for clinical application as biomarkers or therapeutics for a variety of diseases [101]. Perhaps miRNAs that repress CYP450 enzymes to enhance tumor growth could be used as biomarkers if homeostatically their levels vary with concentration or activity of CYP450 induced by exogenous methods. For example, hsa-miR-577, hsa-miR-1, hsa-miR-532-3p and hsa-miR-627, could be used as a biomarker to gauge response to any therapeutics involving CYP3A4. Similarly miR-27b could be used as a biomarker for therapeutics related to CYP1B1 and miR-34a for CYP3A4 and CYP2C19 in addition to transcription factors and other proteins that indirectly affect CYP450 levels or activity (e.g., AKR1D1 and SLC10A1). Conversely, perhaps miRNAs that are endogenously downregulated to increase levels of CYP450 enzymes (thereby enhancing tumor growth) could be used as therapeutic options in various cancers. For instance, exogenous miR148a could be administered to boost its levels and effect on CYP3A4 and CYP2C19 in GI tumors, head & neck cancers, breast cancer, lung cancer and melanoma [80].

#### 4.4.7. Small RNA Therapeutics

In addition to miRNA, the entities antisense oligonucleotides (ASOs), aptamers, siRNAs and synthetic mRNAs are collectively termed small RNAs and are used in the development of RNA interference therapeutics [102]. ASOs are single-stranded deoxyribonucleotides, which bind complementary mRNA targets [103]. This leads to cleavage of the mRNA-DNA heteroduplex by RNase H endonuclease, which prevents translation of the target mRNA and thereby downregulates expression of the corresponding target protein [103]. Aptamers are single-stranded oligonucleotides that bind target mRNA with high affinity and specificity, through physiochemical mechanisms such as hydrophobic, electrostatic, hydrogen bonding, van der Waals forces, base stacking and shape complementarity interactions [104,105]. Due to their desirable tissue penetrability and high affinity and specificity target-binding, aptamers are expected to become a widely used platform for delivery of therapeutic small RNAs [105]. Synthetic small interfering RNAs (siRNAs) are short double-stranded RNAs that contain a guiding strand that binds target mRNA more completely than miRNA and similarly limit translation of the target mRNA [106].

Small RNAs are currently being investigated for RNA-targeting therapeutics to treat (or prevent) illness by limiting translation of mRNA destined to become disease-relevant proteins [102]. Downregulation of disease-causing genes, that is, gene silencing, occurs via binding of the RISC complex to endogenous mRNA. Small RNA can be introduced into the cell via viral vectors or by directly insertion into cytoplasm, where the antisense siRNA-RISC complex then forms and blocks translation of target complementary mRNA. The direct interaction of these small RNA (in the RISC complex) with endogenous mRNA vastly expands the repertoire of possible therapeutics for previously ‘undruggable’ targets. Traditionally, drugs have been developed based on their interactions as ligands for proteins, particularly those with enzyme binding sites [107]. Expanding drug development to small RNAs opens up a new frontier in therapeutics targeting nucleic acids instead of proteins, with potential for overcoming barriers to treating previously intractable diseases [102]. The promise of small RNAs therefore may address close to 85% of the human proteome lacking ligand-binding domains or enzyme binding sites [108,109]. Development of small RNA therapeutics is not without challenges. For decades, development of ASOs and siRNAs has been tempered by immunogenicity, limited potency and poor focused delivery to the cytoplasm of the right cells and tissue [102]. Other setbacks for small RNA therapeutics have included unintended off-target effects on homologous RNA sequences and premature metabolism and excretion of the small RNA. Dozens of trials have been designed to addresses these limitations. While progress is being made to reduce these barriers, more research is needed for application of siRNA therapies to become more widespread [110]. The Food and Drug Administration (FDA) has begun to approve small RNAs as biomarkers to gauge response to therapy or as therapeutics specifically targeting previously undruggable targets, such as the RAS oncogene, with ramification for patients with lung and pancreatic cancer. This is only the beginning of a new era.

#### 4.4.8. Integration of ‘Omics’

In summary, multiple ‘omics’ systems within the individual operate independently and synergistically to modulate various states of health and disease. While it has been known for some time that variations in genomics incompletely account for interindividual variability and genotype-phenotype discordance in P450 enzyme expression, the underlying mechanisms have been unclear. The effect of multiple ‘omics’ on various states of health and disease is slowly being elucidated with continued investigation. The application of systematic multi-omics approaches to precision medicine and systems biology has great potential to improve the care of patients in Cardio-Oncology.

## 5. Clinical Implementation

### 5.1. Pharmacogenomics

Interindividual variability is largely heritable [1,2,7,69,111], thus stimulating interest in utilizing personalization tools such as pharmacogenomics (i.e., the impact of genome variations on individual response to therapeutics) in patient care [2,7,112,113,114,115,116].

#### 5.1.1. Master Regulators

Studies identifying the polymorphisms of “master regulator” genes (affecting multiple CYP enzymes) have revealed key insights, as follows [16]. The 3′-untranslated region (UTR) of the aldo-keto reductase 1D1 (AKR1D1) gene is significantly associated with mRNA expression and enzyme activity of CYP2B6, CYP2C19, CYP2C8 and CYP3A4; the AKR1D1 SNP (rs1872930) yields higher constitutive mRNA expression [16]. Identifying such master regulator genes and categorizing notable SNPs may help clinicians and pharmacists predict levels and activity of CYP450 enzymes and corresponding therapeutic consequences. As CYP450 enzymes metabolize a significant majority of medications, there is a potential role for pharmacogenomics to optimize each individual’s therapeutic response and prevent adverse effects.

#### 5.1.2. Warfarin

To illustrate, a notable example in precision cardiovascular medicine is CYP2C9 genotype-guided dosing of the commonly used anticoagulant warfarin [112,117]. The Clinical Pharmacogenetic Implementation Consortium (CPIC) is an initiative focused on integrating pharmacogenomics into routine clinical care. The consortium has provided guidelines recommending the use of pharmacogenetic dosing algorithms to assist with warfarin dose based on CYP2C9 and VKORC1 genotypes, with consideration of clinical factors. Specifically, CYP2C9 alleles *2 and *3 in addition to *5, *6, *8 and *11 are associated with lower warfarin dosing requirements, due to decreased clearance of the S-enantiomer of warfarin [117,118]. While more is known about alleles *2 and *3, alleles *5, *6, *8 and *11 occur more frequently in the African American populations [117,119]. In spite of these guidelines, at present, patient dosing across much of clinical practice is guided by a dose—response —adjust cycle.

Typically, an empiric dose of warfarin is administered and patient response is tracked by measuring the international normalized ratio (INR). The dose is subsequently adjusted to fit a target threshold depending on the indication for anticoagulation. This may be, at least in part, due to the seemingly inconsistent results of various studies examining the potential impact of genetic testing to guide warfarin dosing. For example, the EU-PACT, GIFT and COAG studies are studies all investigating the utility, efficacy, cost and benefit of genetic testing to guide warfarin dosing decisions [112,117,118,120,121]. The two former studies were conducted largely in homogenous populations (>90% European ancestry), whereas the COAG study was conducted in a more diverse population in the US (28% of trial participants were African American) and did not show a great benefit to warfarin pharmacogenomics [117,120]. However, while EU-PACT administered a loading dose (according to American College of Chest Physicians guidelines) upon initiation of warfarin, the COAG study did not [121,122,123]. Warfarin-related variants have been less studied among African and Hispanic populations, which is notable as there is higher dose variability in these ethnic populations [112,117]. To date, no study has accounted for CYP2C9 variants more common in African Americans (*5, *6, *8, *11). It is unclear whether genetic samples currently in use for warfarin pharmacogenomics accurately represent a diverse US population. The generalizability of these trials may therefore be limited. While guidelines of governing clinical bodies at best recommend weak support of pharmacogenomic testing of warfarin dosing (Class IIB recommendation for primary stroke prevention by the American Heart Association) [124], a small study has shown promise (efficient achievement of therapeutic anticoagulation, fewer supratherapeutic INR values and a shorter duration of low molecular weight heparin) using genotype-guided dosing [117,125]. Further investigation is required.

#### 5.1.3. Clopidogrel

To further illustrate this the potential impact of pharmacogenomics in precision medicine, some individuals with specific CYP2C19 gene mutations are poor metabolizers of the clopidogrel prodrug (the most commonly used antiplatelet agent), which can limit safety of coronary stents after percutaneous coronary intervention [7]. Some studies have demonstrated the possible cost-effectiveness of genotype-guided antiplatelet strategy [126,127,128,129], underscoring the potential utility of precision medicine to tailor medication regimens and achieve optimal patient care. In the genotype-guided antiplatelet strategy, antiplatelet utilization following acute coronary syndrome (ACS) and percutaneous coronary intervention (PCI) would be determined based on CYP2C19 genotype obtained prior to the procedure.

The desire to incorporate genotyping in routine clinical care arose from reports of clopidogrel resistance due to polymorphisms in CYP2C19 [7,117]. The CYP2C19 allele primarily responsible for decreased clopidogrel metabolism is CYP2C19*2. Additionally, multiple alleles encoding deficient enzyme activity can manifest as a true loss-of-function and an inability to metabolize the clopidogrel prodrug. Recently, results of the POPular Genetics trial—a multicenter randomized, open label study of almost 2500 patients based in the Netherlands—demonstrated noninferiority between performing genetic testing for clopidogrel resistance prior to clinical use and using another P2Y12 inhibitor (e.g., ticagrelor or prasugrel) among patients undergoing PCI [130]. As clopidogrel (available as a generic medication) is the least expensive P2Y12 inhibitor, with the lowest bleeding risk [131,132], these results represent a promising option to reduce rates of clopidogrel treatment failure, a boon particularly to those with limited financial resources. TAILOR-PCI is a currently ongoing trial, investigating the effect of the knowledge of genotype to help guide choice of P2Y12 inhibitor In one arm of the trial prospective genotyping is pursued, with individuals possessing loss-of-function (LOF) mutations receiving an alternative P2Y12 inhibitor (e.g., ticagrelor). In the control or conventional care arm, all patients received clopidogrel after PCI and their genotype is only obtained 12 months later after completing therapy. The results of this trial could shed more light on the effect of precision medicine on patient outcomes.

On a population level, CYP450 enzyme polymorphisms can account for part of the variations in drug response common among different ethnic groups. For example, 20% of Asians are poor metabolizers of CYP2C19 (responsible for metabolizing clopidogrel, as well as phenytoin/phenobarbital, omeprazole and so on), while 7% of whites are poor metabolizers of CYP2D6 (responsible for metabolizing beta-blockers, antidepressants, opioids and so on) [1,2,69]. Discussion of CYP450 variations between ethnic groups also underscores the disparities in current literature. For example, while CYP2C9 variants are more frequent in populations of African ancestry, this population is infrequently included in clinical trials [112].

It is important to note that genetic polymorphisms in CYP2C19 do not cause clopidogrel resistance in a vacuum. Comprehensive interactions among pharmacogenomics, patient characteristics and other factors affecting the activity of P450 enzymes largely determine the level of platelet response to inhibition [7]. In addition to the presence of two or more CYP2C19 LOF alleles, type 2 diabetes and increased body mass index (BMI) are likely the most important risk factors, as they independently predict and synergistically contribute to clopidogrel resistance [7,133]. Other important modifiers include chronic kidney disease, hyperlipidemia and age > 65. Lifestyle factors can either potentiate (e.g., diet, caffeine and smoking) or interfere with (e.g., grapefruit) platelet response to clopidogrel, due to interactions with CYP450 enzyme activity [7,133]. Interestingly, a large study in patients with advanced solid tumors showed a 14-fold variation in CYP3A activity, not entirely explained by genetic polymorphisms alone [134]. Potential etiologies include increased chronic inflammatory response ((IL)-1β, TNF-α and IL-6 and cytokine activity), as well as decreased liver function [3,135,136]. This differential CYP450 enzyme activity contributes to interindividual variation, medication bioavailability and therapeutic, null or toxic effects of medications.

### 5.2. Modified P*3 Pathway

An illustration of the optimal interactions of these myriad patient characteristics with the P450 enzyme system is the P*3 pathway [78] (Figure 3). The individual elements of the P*3 pathway—P1, Pre-empt; P2, Predict; P3, Prevent—represent a systems-based approach to patient care. In the pathway, a patient first receives precision counseling, which is akin to genetic counseling but involves digestible information about a suite of tests in precision medicine that can coalesce to form a comprehensive risk assessment. For example, after receiving precision counseling, a patient with breast cancer may in the future receive test results that include a high-risk genetic profile—mutations in TOP2A/B (mediate response to chemotherapy), RAC2 (associates with acute cardiotoxicity phenotype) and NCF4 (associates with chronic cardiotoxicity phenotype) [137]. Systems-based techniques could integrate these separate pieces of information via mathematical modeling into a single risk factor profile accessible by clinicians to help guide the patient towards optimal therapy while minimizing cardiovascular risk [78]. This information would be made available to the patient’s oncologist, cardiologist and primary care provider, all of whom ideally use the same electronic medical record (EMR). With shared decision-making, prior to chemotherapy, her cardiologist may recommend cardioprotective measures with potential clinical utility, such as prophylactic angiotensin converting enzyme inhibitor, statin, beta-blocker or dexrazoxane administered prior to each course of doxorubicin. Her oncologist can also take additional precautions, including using liposomal doxorubicin preparations, avoiding concurrent trastuzumab and anthracycline use or considering alternative therapies [78]. Patient data can then be fed back into the predictive computational models to ensure continuous learning to identify actionable items for implementation in precision Cardio-Oncology. Further, patients at high versus low risk for toxicity from various anti-neoplastic agents (e.g., tyrosine kinase inhibitors, anthracyclines, monoclonal antibodies, immunotherapies) and responders versus non-responders to cardioprotective therapy, could be stratified and identified [137].

In the case of differential metabolism, genomic and other variation leading to altered P450 enzyme activity potentially resulting in drug toxicity may serve as a single hit along the path to cardiotoxicity. The presence of patient characteristics and baseline cardiovascular risk factors or existing cardiovascular disease can increase the risk for cardiovascular toxicity. When a patient’s genotype, drug exposure and other factors accumulate, such multiple hits can further increase the risk of drug toxicity and consequent cardiovascular toxicity [137,138]. Using precision medicine to, for example, uncover known causes of cardiomyopathy (e.g., Titin-truncation mutations) or other cardiovascular diseases in phenotypically normal individuals, may alert patients’ cardiologists and oncologists to take measures to avoid incurring additional hits. In addition to consideration of prophylactic use of ACE inhibitors, statins, beta-blockers or dexrazoxane prior to each course of doxorubicin, other cardioprotective measures may include liposomal doxorubicin preparations, avoiding multiple cardiotoxic regimens or discussion of alternative therapies, thus potentially preventing cardiotoxicity [78,139].

Data from the entire genome can potentially eventually be combined with information about a patient’s transcriptome, proteome, methylome, microbiome, metabolome, environmentome, mutanome, interactome and so on in the P*3 pathway to potentially facilitate delivery of the right therapy to the right patient or group of patients at the right time [112]. Advances in the fields of Cardio-Oncology, precision medicine and Information Technology are therefore coalescing to create new possibilities for prevention and management of cardiac dysfunction from cancer therapy-related adverse effects. Cardioprotection in the oncologic patient involves initiation of cardiac medications in order to minimize or treat cardiotoxicity from cancer therapies, while maximizing administration of indicated cancer treatment. These medications—beta-blockers, angiotensin converting enzyme inhibitors or angiotensin receptor blockers and statins—comprise the cornerstone of cardiovascular disease risk management and treatment in the general population as well as in Cardio-Oncology [140,141]. Perhaps using systems-based approaches in precision medicine to guide the use of these cardioprotective therapies is the panacea of prevention in Cardio-Oncology—in Preventive Cardio-Oncology.

Precision Cardio-Oncology is thus a burgeoning field that seeks to further personalize the cardiovascular care of patients in oncology for decisions related to both management and prevention of cardiovascular toxicities [142]. The goal is to achieve a maximal amount of indicated chemotherapy administered with minimal interruption, while avoiding toxicity. Other notable goals include delivering more effective and efficient care, reducing patient harm and limiting healthcare costs from inappropriate treatment [43]. Indeed, precision medicine has great potential in the care of patients taking medications metabolized by CYP450 enzymes, not only in Cardio-Oncology but in all fields of medicine.

## 6. Conclusions

Metabolism by CYP450 enzymes can determine the bioavailability and thereby efficacy of several drugs in Cardiology and Oncology and in the emergent field of Cardio-Oncology. These enzymes can also affect drug-drug interactions between Oncology and Cardiology drugs. This can compound the use of cardiology drugs for protection from or treatment of cardiovascular toxicity. Differential metabolism of each drug can determine to a certain degree unpredictable bioavailability of the drugs in a specific individual. This can be further impacted by variations in the genome, in the context of the broader epigenome, transcriptome, proteome, microRNA regulome, microbiome, metabolome, environmentome, populome and other components of the individual as a whole organism or system, with multiple parts that can be perturbed by various cardiology or oncology drugs. Increasing knowledge and implementation of the multidimensional impact of endogenous regulatory systems on CYP450-mediated drug metabolism may help preempt drug-drug interactions, predict variations in CYP450 enzymes and prevent complications from subtherapeutic or supratherapeutic drug levels. Such a systems-based view should be considered as we move towards clinical and research practice of Precision Cardiovascular oncology, with particular attention to the role of CYP450 enzymes.

## Figures and Tables

**Table 1:** Most common cytochrome P450 (CYP450) enzymes in humans.

Enzyme	Upper Limit of Normal Percentage of Total Hepatic CYP450 (%)
CYP3A4	37
CYP3A5	1
CYP2C9	29
CYP1A2	16.3
CYP2A6	14
CYP2B6	5.3
CYP2D6	4.3
CYP2C19	3.8

Note: As this is a range, values do not completely sum to 100%. Adapted from [1] with permission.

**Table 2:** A list of CYP450 enzymes and many of their cardiac substrates, inducers and inhibitors.

Enzyme	Substrate Characteristics	Examples of Drugs Relevant to Cardiovascular Care	Inhibitors	Inducers
**CYP3A4 and CYP3A5**	Large & lipophilic molecules, with very diverse structures; includes over 50% of all clinically used drugs CCBs Statins Taxanes (paclitaxel and docetaxel) Sorafenib Dasatinib Cyclophosphamide Proteosome inhibitors (e.g., Bortezomib) Everolimus Cytarabine Dabrafenib Vemurafenib Irinotecan Imatinib/Ibrutinib	Antiarrhythmics: quinidine-3-OH (not 3A5) Calcium Channel Blockers: amlodipine, diltiazem, felodipine, lercanidipine, nifedipine2, nisoldipine, nitrendipine, verapamil HMG CoA Reductase Inhibitors: atorvastatin, cerivastatin, lovastatin, NOT pravastatin, NOT rosuvastatin, simvastatin Propranolol Cilostazol Eplerenone Fentanyl Lidocaine Others: Ondansetron Caffeine-trimethyluric acid Sorafenib	Strong: (Protease Inhibitors) indinavir, nelfinavir, ritonavir, saquinavir, (Antibacterials): clarithromycin, erythromycin, telithromycin; chloramphenicol (Antifungal): itraconazole, ketoconazole, fluconazole, voriconazole (Antidepressant): nefazodone (Vasopressin antagonist): conivaptan Moderate: (Antiemetic): aprepitant (Antibacterials): erythromycin, (Antifungal): fluconazole, (nDP-CCB): verapamil, diltiazem. Mibefradil (Immune modulating agents): Cyclosporine, Tacrolimus (Tyrosine kinase inhibitors -TKIs): Nilotinib, Iapatinib (Hormonal agents): Enzalutamide, Bicalutamide (Chemotherapy): Sorafenib (Misc.): grapefruit juice, starfruit Weak: (H2 blockers): cimetidine (Topoisomerase inhibitors): Etoposide (Anthracyclines): Idarubicine (Alkylating agents): Cyclophosphamide (Antibacterials): ciprofloxacin, norfloxacin (Antifungal): voriconazole, ketoconazole, itraconazole, posaconazole, fluconazole (NNRTI): delavirdine (Antiarrhythmics): amiodarone (SSRIs): fluvoxamine, norfluoxetine (Protease Inhibitors): boceprevir, telaprevir (OCP): * gestodene, mifepristone (Chemotherapy): imatinib (Misc.): starfruit	(NNRTI): efavirenz, nevirapine, efavirenz/emtricitabine/tenofovir (GABA-Agonists): barbiturates, phenobarbital, (Anti-epileptics): carbamazepine, oxcarbazepine, phenytoin (Non-steroidal Anti-androgen): enzalutamide (Antibiotics): rifabutin, rifampin (Misc.): glucocorticoids, modafinil, St. John’s Wort (Thiazolidinedione): pioglitazone troglitazone (Antimitotic agents): Paclitaxel (TKIs): Vemurafenib, Dabrafenib (Hormonal agents): enzalutamide (Angiogenesis inhibitor): Thalidomide (BRAF inhibitor): Vemurafenib
**CYP2C9**	Relatively large and weakly acidic molecules; includes antimalarials and oral antidiabetics Fluvastatin Nateglinide phenytoin-4-OH2 rosiglitazone	Angiotensin II Blockers: losartan irbesartan, valsartan Torsemide S-Warfarin Fluvastatin Rosiglitazone Others: NSAIDs, Sulfonylureas	Strong: fluconazole2 Moderate: amiodarone (NNRTI): efavirenz (Fibrate): fenofibrate (Antifungal): fluconazole, voriconazole (Statin): fluvastatin, lovastatin (SSRI): fluvoxamine2, paroxetine, sertraline (Antibiotic): isoniazid, metronidazole * phenylbutazone, sulfamethoxazole * sulfaphenazole, (Chemotherapeutic): teniposide, 5-flourouracil (Leukotrieine receptor antag LTRA): zafirlukast	(Non-steroidal Anti-androgen): enzalutamide (~3A4/5/7, 2C19) (NNRTI) Nevirapine (Antibiotics): Rifampin (Antiepileptics): phenobarbital, * secobarbital, carbamazepine (Misc.): St. John’s Wort (~3A4,5,7)
CYP2C8	Relatively large and weakly acidic molecules; includes antimalarials and oral antidiabetics Docetaxel Imatinib/Ibrutinib	Torsemide Cerivastatin Amiodarone (n) Thiazoladinedione (Pioglitazone, Rosiglitazone) Others: Repaglinide	Strong: gemfibrozil Moderate: trimethoprim (Thiazolidinediones): glitazones, (LTRA): montelukast (Plant flavonoid): quercetin (found in fruits, vegetables, leaves and grains; red onions and kale)	Rifampin
CYP2E1	Small, generally neutral and hydrophilic, planar molecules; includes aliphatic alcohols and halogenated alkanes Cisplatin	Ethanol	Disulfiram	Ethanol Isoniazid
**CYP1A2**	Planar, aromatic, polyaromatic and heterocyclic amides and amines	Caffeine Naproxen Ondansetron	Strong: fluvoxamine, ciprofloxacin Moderate: Vemurafenib Weak: cimetidine amiodarone, efavirenz, fluoroquinolones, fluvoxamine, furafylline1, interferon, * methoxsalen, * mibefradil, ticlopidine	(Food): broccoli, brussels sprouts, char-grilled meat (AEDs): carbamazepine (Diabetic meds) insulin (Misc.): Modafinil (Antibiotic): Nafcillin, Rifampin (PPI): Omeprazole (Toxins): tobacco
CYP2A6	Nonplanar low molecular weight molecules usually with 2 hydrogen bond acceptors; includes ketones and nitrosamines			
**CYP2D6**	Basic molecules with protonatable nitrogen atom(4–7) Å from the metabolism site; includes many plant alkaloids and antidepressants Proteosome inhibitors	Ondansetron	Strong: bupropion, cinacalcet, fluoxetine, paroxetine, quinidine Moderate: duloxetine, sertraline, terbinafine, sorafenib Weak: amiodarone, cimetidine (NSAID): celecoxib (Antihistamine): chlorpheniramine, * clemastine, diphenhydramine, doxepin, histamine H1 receptor antagonists, hydroxyzine, promethazine, tripelennamine (Antipsychotic): chlorpromazine (SSRI): citalopram, escitalopram (TCA): clomipramine (ChemoRx): doxorubicin, imatinib (Antimalarial): halofantrine (Antipsychotic): haloperidol, levomepromazine, perphenazine (Opioids): methadone (DA agonist, Prokinetic): metoclopramide * mibefradil (Vasopressor): midodrine * moclobemide (H2 blocker): ranitidine (protease inhibitor): ritonavir (Antiplatelet): ticlopidine (Misc.): cocaine	Dexamethasone Rifampin
CYP2B6	Neutral or weakly basic, mostly lipophilic non-planar molecules with 1 to 2 hydrogen bond acceptors; includes anesthetics, insecticides and herbicides cyclophosphamide	N/A	Antiplatelets: clopidogrel, ticlopidine2, Antifungal: voriconazole, Chemotherapeutic: thiotepa	Artemisinin (AED): Carbamazepine, Phenobarbital, Phenytoin (NNRTI): Efavirenz, Nevirapine Rifampin (induces every listed CYP enzyme except 2E1)
**CYP2C19**	Neutral or weakly basic molecules or amides with 2 or 3 hydrogen bond acceptors; includes most proton pump inhibitors Proteosome inhibitors cyclophosphamide	Clopidogrel Labetalol Propranolol R-warfarin→8-OH Others: PPIs: Esomeprazole Lansoprazole Omeprazole2 Pantoprazole	(PPIs): esomeprazole, lansoprazole, omeprazole2, pantoprazole (Antibiotic): chloramphenicol, isoniazid (Antifungal): ketoconazole, voriconazole (H2 blocker): cimetidine (SSRI): fluoxetine, fluvoxamine (NSAID): indomethacin (Dopaminergic): modafinil oral contraceptives (Antiepileptics): oxcarbazepine topiramate (Antiplatelet): ticlopidine (Chemotherapy): sorafenib (Misc.): probenecid	(AED): carbamazepine (NNRTI): efavirenz (Protease Inhibitor): ritonavir (Non-steroidal Anti-androgen): enzalutamide (~3A4/5/7, 2C9) (NNRTI) (OCP): norethindrone (Misc.): prednisone, St. John’s Wort (~3A4/5/7, 2C9) (Antibiotics): Rifampicin

Note: for medications not categorized as strong, moderate or weak inducers/inhibitors, there is insufficient evidence to further categorize them. Medications denoted with an asterisk (*) are not available in the US. Enzymes in bold denote the most commonly occurring CYP450 enzymes. Adapted from various sources [1,2,27,28,29,30,31,32,33,34,35,36,37,38,39,40,41]; used with permisson of the three primary sources [1,2,27].
